# Identification of the Metabolic Enzyme Involved Morusin Metabolism and Characterization of Its Metabolites by Ultraperformance Liquid Chromatography Quadrupole Time-of-Flight Mass Spectrometry (UPLC/Q-TOF-MS/MS)

**DOI:** 10.1155/2016/9240103

**Published:** 2016-09-06

**Authors:** Xianbao Shi, Brianna Mackie, Gang Zhang, Shuman Yang, Yonggui Song, Dan Su, Yali Liu, Lina Shan

**Affiliations:** ^1^Department of Pharmacy, The First Affiliated Hospital of Jinzhou Medical University, Jinzhou, China; ^2^Department of Medicinal Chemistry, Virginia Commonwealth University, Richmond, VA, USA; ^3^Department of Internal Medicine/Community Health Sciences, University of Manitoba, Winnipeg, MB, Canada; ^4^Jiangxi University of Traditional Chinese Medicine, Nanchang 330004, China

## Abstract

Morusin, the important active component of a traditional Chinese medicine,* Morus alba *L., has been shown to exhibit many vital pharmacological activities. In this study, six recombinant CYP450 supersomes and liver microsomes were used to perform metabolic studies. Chemical inhibition studies and screening assays with recombinant human cytochrome P450s were also used to characterize the CYP450 isoforms involved in morusin metabolism. The morusin metabolites identified varied greatly among different species. Eight metabolites of morusin were detected in the liver microsomes from pigs (PLMs), rats (RLMs), and monkeys (MLMs) by LC-MS/MS and six metabolites were detected in the liver microsomes from humans (HLMs), rabbits (RAMs), and dogs (DLMs). Four metabolites (M_1_, M_2_, M_5_, and M_7_) were found in all species and hydroxylation was the major metabolic transformation. CYP1A2, CYP2C9, CYP2D6, CYP2E1, CYP3A4, and CYP2C19 contributed differently to the metabolism of morusin. Compared to other CYP450 isoforms, CYP3A4 played the most significant role in the metabolism of morusin in human liver microsomes. These results are significant to better understand the metabolic behaviors of morusin among various species.

## 1. Introduction

**Figure 1:**
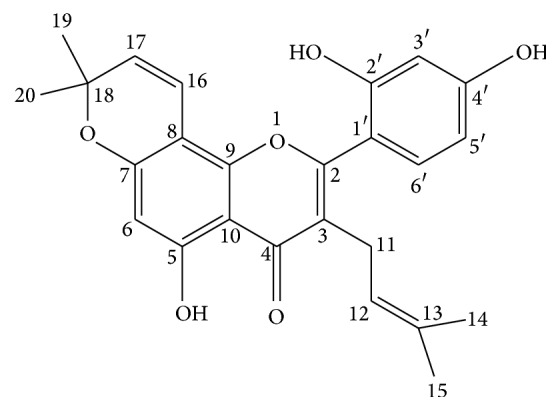
The structure of morusin.

Flavonoids and their derivatives are important components of the human diet and exhibit many diverse pharmacological effects [1, 2]. Morusin (Figure 1) is a prenylated flavonoid that was first isolated from the root bark of* Morus alba L.* in 1978 and then later characterized in 2001 [3, 4]. In previous studies, we discovered that morusin exhibits strong inhibitory activity against primary UGTs and CYP450s. Additionally, our findings revealed significant differences in its metabolism between species and found that dog models are the optimum model to study morusin metabolism [5].

Like most flavonoids, morusin has antitumor [6, 7], anti-inflammatory [8], and antibacterial [9] activity. However, it is unclear if morusin or its metabolites produce these biological activities. Additionally, the side effects and toxic reactions of morusin may also be related to its metabolites. Therefore, determination of only the parent drug may not fully reflect its true pharmacologic and toxic nature. Identification of metabolites of a potential drug candidate plays an important role in drug development and can provide essential information on drug efficacy and its toxicological profile. Recently, metabolic studies have been performed in the early stages of drug discovery to determine whether the potential drug candidate is worth further development [10].

A simple and effective analytical method to reveal the structures of possible active constituents is a significant and valuable tool. Mass spectrometry coupled to liquid chromatography (LC-MS) [11, 12], mass spectrometry coupled to gas chromatography (GC-MS) [13, 14], and nuclear magnetic resonance (NMR) [15, 16] are most frequently used to identify the structures of drugs. Compared to GC-MS or NMR, LC-MS based techniques are often used for metabolite identification due to their superior selectivity, sensitivity, and rapid rate of analysis [12].

In this study, the morusin metabolite profiling was performed through ultraperformance liquid chromatography/electrospray ionization quadruple time-of-flight/high-definition mass spectrometry (UPLC/ESI-Q-TOF/HDMS) combined with pattern recognition approaches and pathway analysis. The system has proven to have higher sensitivity than the traditional high-resolution MS system and has been used to identify many drug structures [17, 18]. In addition, CYP450 isozymes involved in the metabolism of morusin were also confirmed by assays with recombinant CYP450 isoforms and chemical inhibition experiments. Finally, molecular docking was employed to further understand the interactions between morusin and CYP450s.

## 2. Materials and Methods

### 2.1. Materials

Morusin was purchased from Shifeng Corp in Shanghai, China, and its purity was above 98%. Furafylline, xanthotoxin, quercetin, sulfaphenazole, omeprazole, quinidine, clomethiazole, ketoconazole, glucose-6-phosphate dehydrogenase, NADP^+^, D-glucose-6-phosphate, 4-methylumbelliferone (4-MU), 4-methylumbelliferone-*β*-D-glucuronide (4-MUG), Tris-HCl, 7-hydroxycoumarin, and uridine 5′-diphosphoglucuronic acid (UDPGA) (trisodium salt) were purchased from Sigma-Aldrich (St. Louis, MO, USA). Recombinant human supersomes (CYP1A2, CYP2C9, CYP2D6, CYP2E1, CYP3A4, and CYP2C19) were obtained from BD Gentest Corp. (Woburn, MA, USA). All other reagents were of HPLC grade or of the highest grade commercially available.

### 2.2. Preparation and Characterization of Liver Microsomes

Liver microsomes from humans (HLMs), monkeys (MLMs), rabbits (RAMs), rats (RLMs), dogs (DLMs), and minipigs (PLMs) were provided by the Research Institute for Liver Disease Co. (Shanghai, China). The HLMs were prepared from eleven individual human donor livers. MLMs, RAMs, RLMs, DLMs, and PLMs were prepared from ten individual animal livers. Protein concentration and microsome activities of CYP1A2, CYP2A6, CYP2C8, CYP2C9, CYP2C19, CYP2D6, CYP2E1, and CYP3A4 had been previously characterized by the Research Institute for Liver Disease Co.

### 2.3. Analytical Instruments and Conditions

The HPLC conditions have been elaborated by us previously [5]. Briefly, HPLC system (Shimadzu, Kyoto, Japan) consisted of a CBM-20Alite system controller, two LC-20AB pumps, and an SPD-20A ultraviolet light (UV) detector. The chromatographic separation was performed on a C18 column (4.6 mm: 150 mm, 5 mm Kromasil). The mobile phases A and B comprised 0.1% formic acid in water and acetonitrile, respectively. The gradient profiles were as follows: 0–12 min, 55% B; 12-13 min, 55–90% B; 13–19 min, 90% B; 19-20 min, 90–55% B; 20–25 min, 55% B. The flow rate, column temperature, and wavelength were set as 1 mL/min, 40°C, and 270 nm, respectively.

LC-MS/MS analysis was performed on Ultra-Performance Liquid Chromatograph (UPLC) equipped with a Q-TOF SYNAPT G2-Si High Definition Mass Spectrometry (Waters Corporation, Milford, MA). Each sample (2 *μ*L) was injected into a Welch C18 column (1.7 mm × 100 mm, 1.7 *μ*m, Milford, USA) using an Acquity H-class UPLC system (Waters Corporation, USA). The column oven was maintained at 40°C. The mobile phase consisted of LC grade water with 0.1% formic acid (A) and LC grade acetonitrile (B) with the following gradient profile: 0–12 min, 55% B; 12-13 min, 55–90% B; 13–19 min, 90% B; 19-20 min, 90–55% B; 20–25 min, 55% B. The mass spectrometer was operated in negative mode and the mass range was set from 100 *m*/*z* to 500 *m*/*z*. Optimum parameters were as follows: ESI collision gas was Argon, trap collision energies were 6 V (low energy) and 20–50 V (high energy), the capillary and cone voltages were 2 KV and 40 V, the source and desolvation temperatures were 120 and 600°C, and the cone and desolvation gas flows were 50 and 800 L·h^−1^.

### 2.4. Incubation Systems with Liver Microsomes or Recombinant CYP450 Supersomes

The optimum incubation conditions of microsomes have been reported [5]. In brief, the typical incubation system contains 100 mM potassium phosphate buffer (pH 7.4), a NADPH-generating system (1 mM NADP^+^, 10 mM glucose-6-phosphate, 1 unit/mL of glucose-6-phosphate dehydrogenase, and 4 mM MgCl_2_), the appropriate concentration of liver microsomes or recombinant CYP450s supersomes, the corresponding probe substrate, and morusin (or positive inhibitor for different probe reactions) in a final volume of 200 *μ*L. According to preliminary experiments (data not shown), the final protein concentration of 0.3 mg/mL in liver microsomes or 15 nM in recombinant human supersomes and a 30 min reaction time were selected to ensure linear formation of metabolites during the incubations. There was a 3 min preincubation at 37°C before the reaction was initiated by adding the NADPH-generating system. The reaction was placed on ice and terminated by adding 200 *μ*L acetonitrile and an internal standard. The mixture was centrifuged at 20,000 ×g for 20 min and an aliquot (10 *μ*L) of supernatant was transferred for HPLC or LC-MS/MS analysis.

### 2.5. Assay with Recombinant P450s

Six cDNA-expressed human CYP isoforms (CYP1A2, CYP2C9, CYP2C19, CYP2D6, CYP2E1, and CYP3A4) were used to screen the involved isoform(s) for morusin metabolites. The incubations were carried out with each of the CYP450 isoforms using the protocol described above. Morusin (10 *μ*M) was incubated with each of the recombinant CYP450s (15 nM) at 37°C for 60 min and potential metabolites were monitored by HPLC.

### 2.6. Chemical Inhibition Assays

In this study, morusin metabolism in HLMs was measured in the absence or presence of selective inhibitors for CYP1A2, CYP2C9, CYP2C19, CYP2D6, CYP2E1, and CYP3A4 to explore the enzyme(s) involved in the biotransformation of morusin. The positive control inhibitors of individual CYP450 isoforms were as follows: furafylline (10 *μ*M) for CYP1A2, xanthotoxin (2.5 *μ*M) for CYP2A6, quercetin (2 *μ*M) for CYP2C8, sulfaphenazole (10 *μ*M) for CYP2C9, omeprazole (20 *μ*M) for CYP2C19, quinidine (10 *μ*M) for CYP2D6, clomethiazole (50 *μ*M) for CYP2E1, and ketoconazole (1 *μ*M) for CYP3A4.

### 2.7. Docking Studies of Morusin into the Reported Structures of CYP3A4

In order to further assess morusin metabolism by CYP3A4, molecular docking analysis with Gold v5.2 was implemented. This docking method has been reported previously [19]. The crystal structures of human CYP3A4 (pdb: 4K9W) were obtained from RCSB Protein Databank (http://rcsb.org/). SYBYL X2.1 was used for protein and ligand preparation, and energy minimization was completed through the external Tripos force field. The protonation state and energy minimization of the protein and the ligands were calculated using the default settings in SYBYL X2.1. The docked poses were scored using CHEMPLP scoring function. The highest scored docked pose of the ligand was visualized using Pymol Molecular Graphics System v1.3.

## 3. Results

### 3.1. Identifying CYP450s Involved in the Metabolism of Morusin

**Figure 2:**
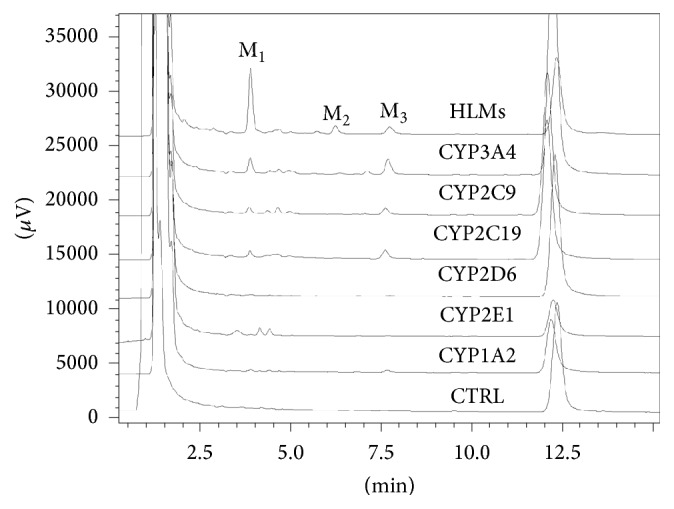
Representative HPLC profiles of morusin and its metabolites in cDNA-expressed human P450 isoforms and liver human microsomes.

**Figure 3:**
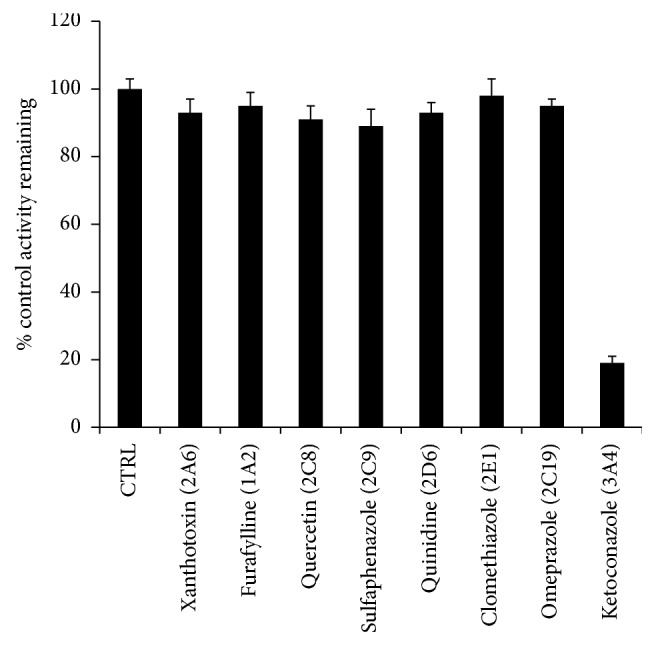
Inhibition assays of morusin metabolism by selective CYP450 inhibitors in HLM. Results were mean ± SEM of at least 3 separate assays.

cDNA-expressed human P450 isoforms were used to investigate the enzymes involved in the formation of M_1_. Quantification of M_1_ in HLMs was normalized to 100% and other enzymes including CYP3A4, CYP2C19, CYP2D6, CYP2E1, CYP2C9, and CYP1A2 were compared with its counterpart from HLM incubation. As shown in Figure 2, the amount of M_1_ generated by CYP3A4, CYP2C19, CYP2D6, CYP2E1, CYP2C9, and CYP1A2 was 22.6%, 6.4%, 0.6%, 1.7%, 5.9%, and 2.1% of the amount incubated in HLMs, respectively. In addition, we also used chemical inhibition assays to confirm the key role of CYP450s in metabolizing morusin. The formation of M_1_ could be potently inhibited by ketoconazole (a potent inhibitor of CYP3A4) to less than 20% activity, while the selective inhibitors of other CYP450 isoforms had minor effects on the formation of M_1_ (Figure 3).

### 3.2. Structure Characterization of Metabolites by LC-MS/MS

**Figure 4:**
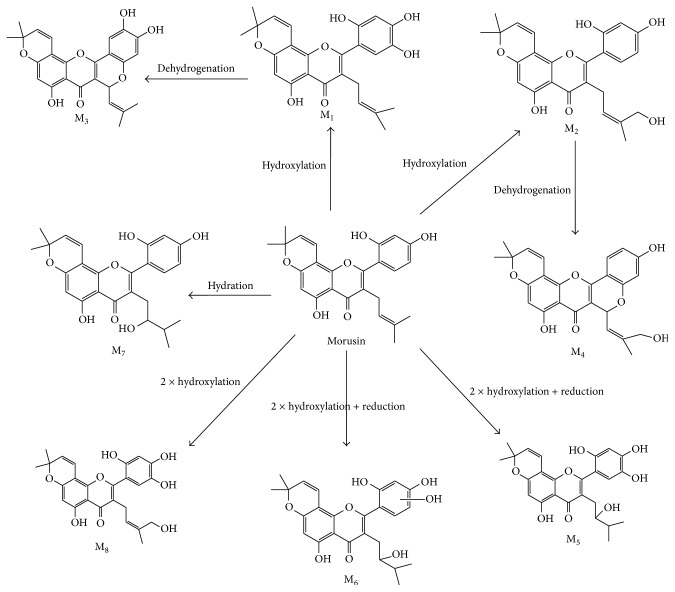
Proposed chemical structures and major metabolic pathway of morusin in six species (including rat, monkey, dog, rabbit, minipig, and human).

**Figure 5:**
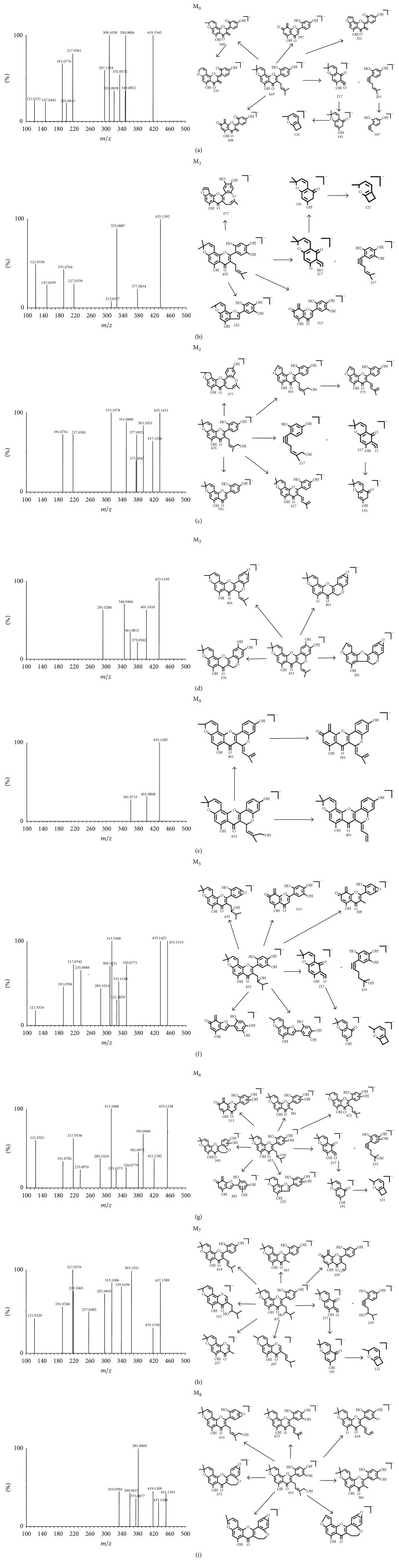
Structural formula and fragmentation pathway of M_0_–M_8_.

In this study, the biotransformation of morusin was investigated by incubating morusin with different liver microsomes. Eight metabolites (M_1_–M_8_) were identified from MS and MS/MS data. All of the morusin metabolites were detected in PLMs, RLMs, and MLMs. Six metabolites were detected in HLMs (M_1_–M_3_, M_5_–M_7_), RAMs (M_1_–M_3_, M_5_, M_7_, and M_8_), and DLM (M_1_, M_2_, and M_4_–M_7_). Morusin metabolite data is listed in Table 1, and the structural skeleton of morusin and its potential metabolic pathways are shown in Figure 4. The parent compound, morusin, had a retention time of 9.17 min and was easily elucidated from the standard by comparison of retention time and MS data. Morusin (*m*/*z* 419.1545) exhibited eleven characteristic fragment ions at *m*/*z* values of 350, 349, 335, 321, 309, 297, 217, 201, 191, 147, and 121 (Figure 5(a)). Fragment ions at *m*/*z* 217.0561 and 201.0641 were produced by retro-Diels-Alder (RDA) cleavage and further fragmented to produce *m*/*z* 191.0776 and 147.0441. The fragment ion at *m*/*z* 191.0776 was produced from parent ion at *m*/*z* 217.0561 through losing a CO and adding two hydrogens at C-10. The fragment ion at *m*/*z* 335.0574 was a daughter ion of parent ion at 349.0852 by losing -CH_2_.

M_1_ and M_2_ were isomers with the same molecular formula (C_25_H_24_O_7_) but different MS/MS and retention times (7.15 and 8.66 min). They showed a *m*/*z* of 16 Da more than morusin indicating that the two metabolites were hydroxylated morusin occurring at C-5′ and C-14, respectively. The cleaved pathways and fragment ions of M_1_ and M_2 _are shown in Figures 5(b) and 5(c). The product ion at *m*/*z* 351.0890 could be generated from a neutral loss (-C_5_H_8_OH) at C-3. The fragment ion at *m*/*z* 377 was a cyclization product from M_1_ or M_2_ occurring at C-14 and C-2′-OH.

M_3_ and M_4_ had the same molecular formula of C_25_H_22_O_7_ and showed a *m*/*z* of 2 Da less than M_1_ or M_2_. According to the cleaved pathways and fragment ions of M_3_ and M_4 _shown in Figures 5(d) and 5(e), they were identified as the cyclization and dehydrogenation products of M_1_ and M_2_, respectively. The cyclized sites of M_3_ or M_4_ occurred at C-11 and C-2′-OH. M_3_ underwent dehydration reaction and further produced the fragment ions at *m*/*z* 291, 361, and 401. Thus, M_3_ was inferred to have a hydroxyl at C-5′. Because M_3_ was produced from M_1_, we inferred M_1 _also had a hydroxyl at C-5′.

M_5_ and M_6_ were also isomers with the same molecular formula of C_25_H_26_O_8_ (*m*/*z* 453.1515) but different retention times. The ionized form of M_5_ and M_6_ was 34 Da more than the parent compound. According to the MS/MS spectra and information shown in Figures 5(f) and 5(g), M_5_ and M_6_ were identified as the reduction and dihydroxylation products of morusin. Exact mass determination and the corresponding chemical composition verify that M_5 _was produced through the addition of two hydroxyl groups to the parent molecule at C-12 and C-5′. M_6_ also has two more hydroxyl groups than morusin: one hydroxyl group was added in phenyl and the other hydroxyl group was added at C-12.

The molecular formula of M_7_ was determined to be C_25_H_26_O_7_ (*m*/*z* 437.1389) based on MS data and its ionized form was 18 Da more than the parent morusin. According to its molecular formula and MS/MS spectra, M_7_ was identified as the hydration product of morusin where the hydroxyl group was bonded to C-12. Structural formula and fragmentation pathway of M_7_ are shown in Figure 5(h).

The ionized form of M_8 _appeared at *m*/*z* 451.1393 and was 32 Da more than morusin. Additionally, corresponding main fragment ions were found at *m*/*z* 433.1366, 419.1509, 381.0969, and 375.0877. Therefore, M_8_ was identified as a dihydroxylation product of morusin. According to the structures of fragment ions, the two new hydroxyl groups are potential bonded to C-14 and C-5′ (Figure 5(i)).

### 3.3. Analysis of Docking Results

**Figure 6:**
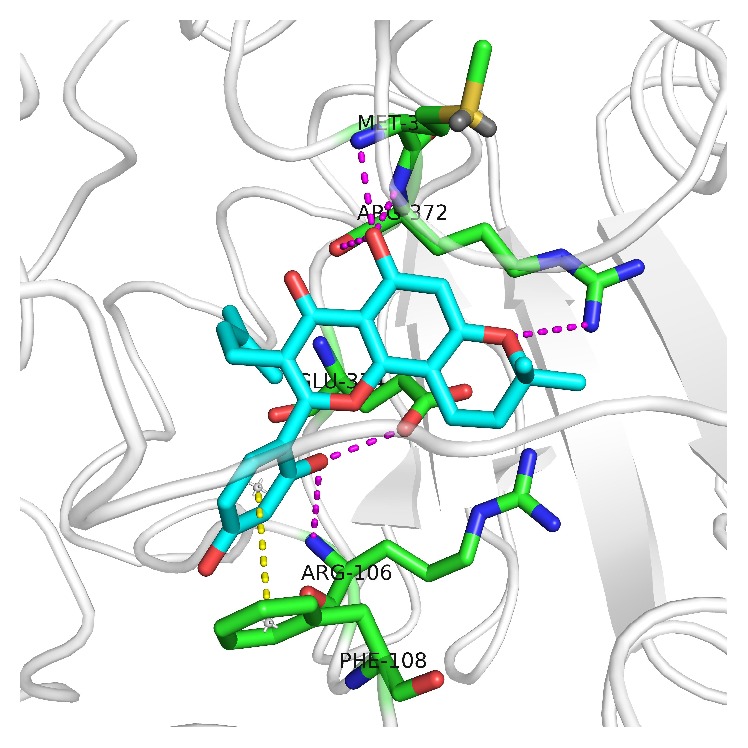
Binding mode of morusin with CYP3A4. Morusin is shown in cyan, hydrogen bonds are displayed in purple dotted lines, and *π*-*π* stacking is displayed in a yellow dotted line.

CYP3A4 is the major enzyme involved in the metabolism of morusin. Therefore, we used molecular docking to study the molecular mechanism of interactions between morusin and CYP3A4. As shown in Figure 6, morusin binds to the active cavity of CYP3A4 through hydrogen bonding and *π*-*π* stacking interactions. The hydrogen bonds occur with Arg106, MET371, Arg372, and Glu374, and *π*-*π* stacking interactions occur with Phe108.

## 4. Discussion

Morusin has many significant properties including antitumor, antibacterial, and anti-inflammatory properties and its pharmacological effects have been studied thoroughly. However, the metabolic pathway and behavior of morusin in human and experimental animals vary greatly and have not been examined. In this study, a comparison of metabolic profiles, enzymes involved, and catalytic efficiency of morusin metabolism in liver microsomes from different species was performed.

It is necessary during a toxicity assessment to identify the enzyme(s) involved in morusin biotransformation in order to predict potential metabolite-drug interactions [20]. We identified CYP450 isoforms involved in morusin metabolism through screening assays with commercially available cDNA-expressed CYP450 isoforms including CYP1A2, CYP3A4, CYP2E1, CYP2C9, CYP2D6, and CYP2C19. Of the six CYP isoforms, CYP3A4 was identified as the enzyme most responsible for M_1_ formation. Nevertheless, each of the six isoforms that catalyze the metabolism of morusin was weaker than when incubated in HLMs. This may be the reason that morusin displayed strong inhibition to CYP450s [5]. In addition, chemical inhibition studies were performed to confirm the CYP450 isoform that catalyzes morusin metabolism and the results indicated that the CYP3A4-specific inhibitor, ketoconazole, potently inhibited the formation of M_1_. Therefore, these results verified that morusin is primarily catalyzed by CYP3A4 in comparison to other CYP450 isoforms. Molecular docking studies also confirmed that morusin binds in the active cavity of CYP3A4 and interacts through hydrogen bonds and *π*-*π* stacking. CYP3A4 has been identified as the important isozyme involved in the metabolism of many flavonoids such as nobiletin [21] and ipriflavone [22].

This study presents the fragmentation pathways of morusin through implementation of LC-MS/MS in negative ion mode and investigates its metabolites and metabolic pathways in six species. The results indicate that eight metabolites of morusin are primarily biotransformed through hydroxylation, reduction, cyclization, dehydrogenation, and dehydration reactions. Flavonoids with remarkable regioselectivity at the benzoannelated pyran ring are often formed through Retro Diels-Alder (RDA) reactions [23]. M_0_, M_1_, M_2_, M_5_, M_6_, and M_7_ were identified as the metabolites that underwent the RDA process. Under the effect of LC-MS/MS, M_3_, M_5_, or M_8_ occurred dehydration reaction in phenyl and produced the corresponding fragment ions. Thus, we inferred that M_3_, M_5_, or M_8_ had a hydroxyl at C-5′. Because M_3_ was the dehydrogenation product of M_1_, M_1_ maybe has a hydroxyl at C-5′. All morusin metabolites were detected in PLMs, RLMs, and MLMs, which was consistent with previous publications which stated that pigs, rats, and monkeys have a greater* capacity* to metabolize morusin [5]. M_1_, M_2_, M_5_, and M_7 _were found in all species, which indicated that hydroxylation was the major metabolic transformation of morusin. This novel* in vitro* morusin metabolism information provided in our investigation is vital to develop a new drug and better understand the safety and efficacy of the drug.

In conclusion, eight metabolites from morusin were observed in the liver microsome incubation system and hydroxylation was the major metabolic transformation. CYP3A4 was the major isozyme involved in morusin metabolism, and molecular docking studies signified that morusin binds to CYP3A4 through hydrogen bonds and *π*-*π* interactions. Identification of the metabolites and metabolic enzymes for morusin is an important contribution to fully understand the pharmacological and toxicological profile of morusin its pharmacological and toxicological study.

## Figures and Tables

**Table 1:** *m*/*z* of [M-H]^−^ measurements for the protonated molecules of metabolites in rat, monkey, pig, rabbit, human, and dog liver microsomes.

Number	*t*_*R*_	Formula	Mass	Fragment	Error (ppm)	Metabolic reaction	Species					
							Rats	Monkey	Pigs	Rabbits	Human	Dog
M_0_	9.17	C_25_H_24_O_6_	419.1545	350, 349, 335, 321, 309, 297, 217, 201, 191, 147, 121	+1.1	Parent	D	D	D	D	D	D
M_1_	8.66	C_25_H_24_O_7_	435.1392	377, 325, 313, 217, 191, 121	+0.2	Hydroxylation	D	D	D	D	D	D
M_2_	7.15	C_25_H_24_O_7_	435.1451	417, 393, 377, 375, 351, 313, 217, 191	−0.2	Hydroxylation	D	D	D	D	D	D
M_3_	7.50	C_25_H_22_O_7_	433.1195	401, 379, 361, 346, 291	−0.1	Hydroxylation + dehydrogenation	D	D	D	D	D	ND
M_4_	7.09	C_25_H_22_O_7_	433.1285	401, 361	+2.2	Hydroxylation + dehydrogenation	D	D	D	ND	ND	D
M_5_	8.82	C_25_H_26_O_8_	453.1515	435, 350, 332, 325, 313, 309, 285, 235, 217, 191, 121	+0.6	2 × hydroxylation + reduction	D	D	D	D	D	D
M_6_	8.10	C_25_H_26_O_8_	453.1528	421, 393, 381, 350, 325, 313, 285, 235, 217, 191, 121	−2.6	2 × hydroxylation + reduction	D	D	D	ND	D	D
M_7_	8.34	C_25_H_26_O_7_	437.1389	419, 365, 339, 315, 297, 257, 219, 217, 191, 121	+1.9	Hydration	D	D	D	D	D	D
M_8_	8.77	C_25_H_24_O_8_	451.1393	433, 419, 381, 375, 360, 333	+0.2	2 × hydroxylation	D	D	D	D	ND	ND

D, detected; ND, not detected.
